# Illicit alcohol consumption and its associated factors among patrons in Zambia: a cross-sectional analytical study

**DOI:** 10.3389/fpubh.2025.1444304

**Published:** 2025-04-24

**Authors:** Cosmas Zyambo, Masauso Moses Phiri, Richard Zulu, Musawa Mukupa, Kumbulani Mabanti, Tulani Francis L. Matenga, Angela Rizzo, Anna Hainze, Ahmed Ogwell, Dhally Menda, Fastone Goma, Tom Achoki

**Affiliations:** ^1^Department of Community and Family Medicine, School of Public Health, University of Zambia, Lusaka, Zambia; ^2^Center for Primary Care Research, Lusaka, Zambia; ^3^Department of Pathology and Microbiology, School of Medicine, University of Zambia, Lusaka, Zambia; ^4^Department of Health Promotion, School of Public Health, University of Zambia, Lusaka, Zambia; ^5^AB InBev Foundation, New York, NY, United States; ^6^Independent Researcher, Nairobi, Kenya; ^7^United Nations Foundation, New York, NY, United States; ^8^School of Postgraduate Studies, University of Zambia, Lusaka, Zambia; ^9^Churches Health Association of Zambia (CHAZ), Lusaka, Zambia; ^10^Africa Institute for Health Policy, Nairobi, Kenya

**Keywords:** illicit alcohol, distilled beverage, traditional alcohol, males, Zambia

## Abstract

**Introduction:**

Illicit alcohol consumption is prevalent globally, particularly in low- and middle-income countries where formal regulatory systems are weak or less enforced. Consumption of illicit alcohol has serious consequences, both immediate and long-term.

**Objective:**

To examine the socio-demographic and behavioral factors associated with the consumption of illicit alcohol among the patrons in selected urban and peri-urban areas in Zambia.

**Methods:**

This was a cross-sectional study. Illicit alcohol consumption status, socio-demographics and behavioral factors were assessed. Adjusted multivariable logistic regression was used to obtain odds ratios (AOR) at a 95% confidence interval (CI).

**Results:**

Of the 416 patrons who participated, 71.2% consumed illicit alcohol (Men, 75.3% vs. women 54.7%). More than 66.8% reported needing a drink first thing in the morning, 50.2% have had problems with friends due to alcohol and 19% did indicate having visited the hospital due to a drinking problem. In multivariable analysis, factors significantly associated with decreased odds of illicit alcohol consumption were females (AOR) 0.38 (95% CI: 0.20–0.73, *p* = 0.003), Ndola city (AOR = 0.28, 95% CI: 0.12–0.62, *p* = 0.002), drinking pattern of 2–3 days a month (AOR: 0.19, 95% CI: 0.06–0.53, *p* = 0.002) and incomes above K10,000 (AOR: 0.40, 95% CI: 0.19–0.85, *p* = 0.017). The inability to stop drinking (AOR = 2.86, 95% CI: 1.22–6.69, *p* = 0.016) had an increased odds of illicit alcohol consumption.

**Conclusion:**

Our findings underscore the high prevalence of illicit alcohol consumption among the patrons. Addressing illicit alcohol consumption requires a multifaceted set of interventions that consider the various factors contributing to alcohol misuse, and focuses on prevention, education, support, and community engagement.

## Introduction

Overconsumption of alcohol is a global health concern, with three million deaths attributed to alcohol use annually (1). It is a risk factor for non-communicable diseases and injuries, and is associated with violence, suicide, child abuse, sickness, and absence from work (2, 3). Globally, alcoholic beverages are classified as either recorded or unrecorded (also known as illicit). Recorded alcohol is controlled, regulated, tracked, produced, and purchased legally. Illicit or unrecorded alcohol, on the other hand, includes products: (1) produced by unlicensed industries/individuals; (2) home brewed/homemade; (3) unregulated; (4) smuggled across borders; (5) consumed in unregistered jurisdiction; (6) not intended for consumption such as surrogate alcohol (e.g., hand sanitizer or aftershave) (4–6). It is estimated that more than 25% of all alcohol consumed worldwide is illicit (3), with Africa consuming illicit alcohol more frequently and in large quantities compared to recorded alcohol (7, 8). Illicit alcohol consumption has become a major public health threat and, because it’s not regulated and is sold illegally without payment of tax or duty (3, 9), it is often the cheapest form of alcohol and is often consumed by the more vulnerable populations who are of low socioeconomic status (SES) (8).

Epidemiological studies have consistently revealed high rates of illicit alcohol use, with variations observed across regions, cultures, and socio-economic backgrounds (9–12). Illicit alcohol consumption encompasses various forms, including underage drinking, binge drinking, and the consumption of counterfeit or illegally produced alcohol (12). Few studies have been conducted in Zambia to generate enough epidemiological data on illicit alcohol. In the general population a STEPS survey-2017 was conducted and showed that 26.3% of the participants interviewed consumed illicit alcohol (13). Mungandi et al. (12), examined the predictors of alcohol consumption among adolescents and young adults in the capital city of Lusaka, finding that 27% consumed low-cost, high- Alcohol by Volume (ABV) manufactured illicit spirits locally known as *junta or tujilijili* and 18% consumed a home-distilled spirit called *kachasu*, both of which are illicit alcohol. A myriad of factors contribute to the prevalence of illicit alcohol consumption (12, 14), these include peer pressure (12), parental attitudes toward alcohol especially in young people (14, 15), ease of access to alcohol (16), socioeconomic disparities (14), mental health issues (17), and exposure to alcohol marketing. Social and cultural norms surrounding alcohol use also play a significant role in shaping people’s drinking behaviors (14).

The health and social implications of recorded alcohol consumption are well established (3, 8, 18), however, there is a paucity of data on illicit alcohol consumption in the sub–Saharan Africa, particularly in Zambia. Illicit alcohol consumption is becoming a huge public health problem in Zambia however there is lack of epidemiological and associated factors on illicit alcohol consumption, this study seeks to fill this knowledge gap. We therefore aimed to examine the socio-demographic and behavioral factors associated with the consumption of illicit alcohol among the patrons in selected urban and peri-urban areas in Zambia. Understanding its epidemiology and associated factors of illicit alcohol consumption is crucial in developing and implementing targeted interventions aimed at reducing its prevalence and mitigating the impact on the people, both at the individual and societal levels.

## Materials and methods

### Study setting, design, and procedures

This is a cross sectional analytical study conducted in three urban and peri-urban areas in Zambia; Livingstone, Lusaka, and Ndola. The selection of the study sites was purposeful and included Lusaka, the capital city of Zambia, and Ndola on the Copperbelt as those were reported to have high consumption of illicit alcohol due to undeclared (tax leakage) production of alcohol and smuggling (19). Livingstone was also purposively sampled as a border city that shares a border with Namibia, Botswana, and Zimbabwe.

**Figure 1:**
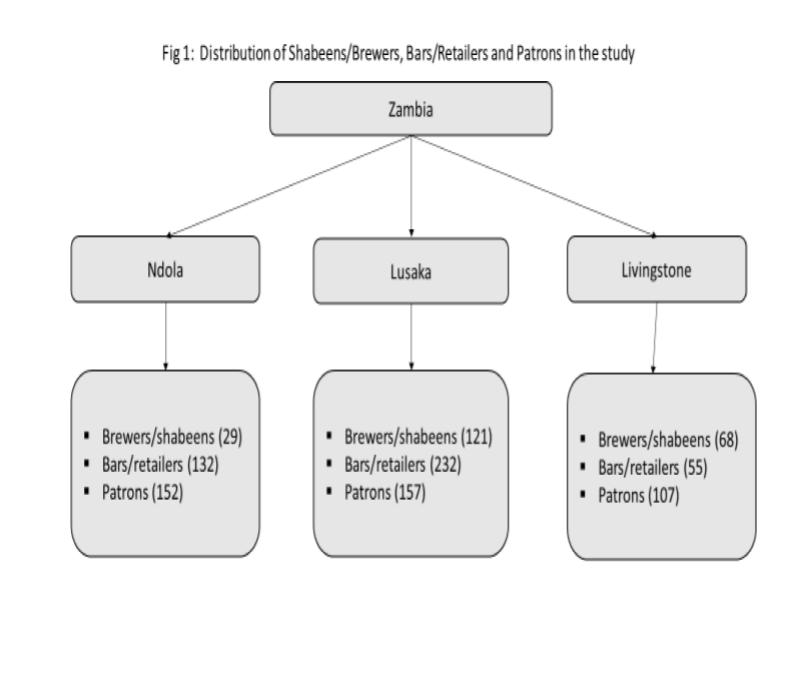
Distribution of shabeens/brewers, bars/retailers and patrons in the study.

To enable us to assess alcohol consumption, especially illicit alcohol in a more representative manner, we adopted the Zambian Ministry of Health’s Level 1 hospital catchment zones and purposefully selected the compounds (township neighborhoods). These compounds are densely populated areas where people live on less than a dollar a day [approximately 27 Zambian Kwacha (K)]. Most residents earn their living by selling merchandize within their neighborhoods. While most bars/retailers are located at the market, there are also shabeens/brewers; neighborhood production/drinking places within these communities. We purposely selected bars/retailers and shabeens/brewers from these compounds for our study (Figure 1). After obtaining consent from the patrons, we scheduled interviews for the following day. These compounds were purposely selected as they are the major compounds in the cities; in Lusaka 121 shabeen/brewers and 232 bars/retailers were purposely selected across the following componds: COMESA market, Matero, Kanyama, Chawama, Chilenje, Mtendere, Kamanga, Bauleni, Chibolya and Chipata. In Ndola, 29 shabeen/brewers and 132 bars/retailers selected across Main market, Main Masala, Chifubu, and Kaloko, and in Livingstone, 68 shabeen/brewers and 55 bars/retailers selected across Town Center (Zimbabwe market), Maramba, Linda, Libuyu, and Dambwa. The data was collected between 4th September and 4th October 2023. Inclusion criteria: (1) major compounds in the city, (2) major brewers (3) main markets, (4) main bars/retailers.

Exclusion criteria: (1) anyone who did not consent.

To calculate the sample size for this study on alcohol consumption in Zambia we used the following formula: *n* = (*Z*^2^ * *p* * *q*) / *E*^2^ Where: *n* = required sample size:

*Z* = *Z*-value corresponding to the desired confidence level (for 95% confidence level, *Z* ≈ 1.96).

*p* = expected prevalence (proportion) of alcohol consumption (20).

*q* = 1 - *p* (complement of the expected prevalence).

*E* = desired level of precision (margin of error).

*n* = (1.96^2^ * 0.207 * 0.793) / 0.05^2^.

*n* ≈ 308. 10% was added to compensate for non-response or other design effects.

Our total sample size for the patrons was 340, proportionately allocated to size [Lusaka (140) and Livingstone and Ndola (100 each)]. The study nevertheless managed to interview a total sample of 416 patrons as it still had enough resources to interview more. The research assistants (RAs) interviewed the patrons before they started drinking alcohol at the bars.

### Data collection and management

Using a modified alcohol use and disorder identification test (AUDIT) segment of the WHO-STEPs survey questionnaire (13), the four trained RAs in each city collected the survey data. The questionnaire included items on socio-demographic characteristics of the participant and alcohol consumption. The modified questionnaire also included the types of alcohol consumed and whether they have consumed any homebrewed alcohol (*ngankata, kachasu*). Data was primarily collected using Tablets with Open Data Collect (ODK). RAs ensured that participants’ anonymity was maintained. The entered data was cleaned/validated and backed up daily on a secure cloud storage system as described on “Data Security” and on an external hard drive.

### Dependent variables

The primary outcome was illicit alcohol consumption. This is study any of the following defined illicit alcohol, (1) high-ABV manufactured illicit spirits locally known as junta or tujilijili, (2) smuggled spirits and (3) home-distilled spirit called kachasu (19). Participants were asked in the questionnaire, “Have you consumed at least one drink of illicit alcohol on any of the past 7 days?” Those who reported “YES” were coded as 1, indicating illicit alcohol consumption while those who reported “NO” were coded as 0, indicating no illicit consumption.

### Covariates

To assess the factors that influence illicit alcohol consumption, several sociodemographic, behavioral and levels of alcohol dependency factors were considered.

*Sociodemographic variables*: Age, sex, employment status; education level, monthly income and marital status.

*Behavioral variables*: frequency of drinking (Every day, 5–6 days a week, 3–4 days a week, 1–2 days a week, 2–3 days a month and about 1 day a month, drinking pattern); Drinking pattern (An occasional drinker, A light drinker, A social drinker, A heavy drinker, and A binge drinker); Age at initiation of drinking (< 15, 15–19, 20–25, > 25), and introducer to drinking (Friend or acquaintance, Brother or sister, Parent, Other relative, stole it, purchased it myself, cannot recall).

*Alcohol dependency variable*, unable to stop drinking once starts (Yes or No), failure to do what was normally expected from you because of drinking (Yes or No), often have you needed a first drink in the morning to get yourself going after a heavy drinking session (Yes or No).

*Social impact variable*, had problems with friends due to drinking (Yes or No).

*Health consequence variables*, visited hospital due to drinking (Yes or No), intent to quit (Yes or No), and ever stopped drinking due to health reasons (Yes or No).

### Statistical analysis

Characteristics of alcohol drinkers were calculated for the overall population and stratified by illicit consumption or not. Continuous variables were reported as the means (SD), and categorical variables were reported as frequencies with percentages. Using logistic regression, univariate and multivariable analyses were conducted to calculate unadjusted and adjusted odds ratios (ORs) and the corresponding 95% confidence intervals (CIs) for the association between the independent variables and the outcome of illicit alcohol consumption. Based on literature, age, marital status, education and age at first drink were determined *a priori* and included in the full model regardless of the univariate statistical significance. The statistical significance level was set at *p* < 0.05 (two-tailed test). All analyses were conducted using STATA version 15.

## Results

### Population and distribution

This study involved 416 patrons living in Livingstone, Ndola, and Lusaka of Zambia, out of which 332 (79.8%) were men and 84 (20.2%) were women. The largest proportion of patrons who consumed illicit alcohol was found in those aged 30–44 (47.8%), living in Ndola (36.6%) and Lusaka (37.7%); and having high school education (40.3%). According to employment status, 86.5% of the patrons were employed with 69.5% of them earning a salary less than K4,001. The age at first drink (45.2%) of these patrons was between 15 and 19 years with 38.7% drinking alcohol every day. Almost 71% reported having intentions to stop drinking. More than 66.8% needed a drink first thing in the morning, 50.2% had problems with friends due to alcohol. (Table 1).

**Table 1:** The characteristics of patrons according to their illicit alcohol consumption among patrons in selected cities of Zambia.

Characteristics	Overall	Illicit alcohol consumption (Yes)	Illicit alcohol consumption (No)
	*N* (%)	*N* (%)	*N* (%)
Socio-demographic	416 (100)	296 (71.2)	120 (28.8)
Age group			
18–29	141 (33.9)	102 (34.5)	39 (32.5)
30–44	199 (47.8)	142 (48.0)	57 (47.5)
45–59	66 (15.9)	46 (15.5)	20 (16.7)
60–69	6 (1.4)	4 (1.4)	2 (1.7)
70 and above	4 (1.0)	2 (0.7)	2 (1.7)
Gender			
Male	332 (79.8)	250 (84.5)	82 (68.3)
Female	84 (20.2)	46 (15.5)	38 (31.7)
Marital status			
Married	183 (44.1)	132 (44.6)	51 (42.9)
Not married	203 (48.9)	140 (47.3)	63 (52.9)
Separated	29 (7.0)	24 (8.1)	5 (4.2)
Cities			
Livingstone	107 (25.7)	91 (30.7)	16 (13.3)
Lusaka	157 (37.7)	106 (35.8)	51 (42.5)
Ndola	152 (36.5)	99 (33.5)	53 (44.2)
Education			
Primary school	73 (17.6)	50 (17.0)	23 (19.2)
Secondary school	110 (26.6)	76 (25.9)	34 (28.3)
High school	167 (40.3)	127 (43.2)	40 (33.3)
College/ University	64 (15.5)	41 (14.0)	23 (19.7)
Employment			
Employed	360 (86.5)	262 (88.5)	98 (81.7)
Unemployed	47 (11.3)	28 (9.5)	19 (15.8)
Student	9 (2.2)	6 (2.0)	3 (2.5)
Income			
Less than 4,001	289 (69.5)	206 (69.6)	83 (69.2)
4,001 – 10,000	79 (19.0)	65 (22.0)	14 (11.7)
Above 10,000	48 (11.5)	25 (8.5)	23 (19.2)
Behavioral variables			
Age at first drink			
< 15	92 (22.1)	64 (21.6)	28 (23.3)
15–19	191 (45.9)	149 (50.3)	42 (35.0)
20–25	94 (22.6)	59 (19.9)	35 (29.2)
> 25	39 (9.4)	24 (8.1)	15 (12.5)
Alcohol consumption			
Every day	161 (38.7)	133 (44.9)	28 (23.3)
5–6 days a week	23 (5.5)	19 (6.4)	4 (3.3)
3–4 days a week	84 (20.2)	63 (21.3)	21 (17.5)
1–2 days a week	101 (24.3)	61 (20.6)	40 (33.3)
2–3 days a month	30 (7.2)	14 (4.7)	16 (13.3)
About 1 day a month	8 (1.9)	1 (0.3)	7 (5.8)
Less often	9 (2.2)	5 (1.7)	4 (3.3)
First alcohol introducer			
Friend or acquaintance	229 (55.1)	172 (58.1)	57 (47.5)
Brother or sister	18 (4.3)	15 (5.1)	3 (2.5)
Parent	33 (7.9)	24 (8.1)	9 (7.5)
Spouse or partner	9 (2.2)	4 (1.4)	5 (4.2)
Other relatives	17 (4.1)	11 (3.7)	6 (5.0)
Stole it	33 (7.9)	20 (6.8)	13 (10.8)
Purchased it myself	62 (14.9)	42 (14.2)	20 (16.7)
Cannot recall	15 (3.6)	8 (2.7)	7 (5.8)
Alcohol pattern			
An occasional drinker	51 (12.3)	31 (10.5)	20 (16.7)
A light drinker	149 (35.8)	108 (36.5)	41 (34.2)
A social drinker	108 (26.0)	71 (24.0)	37 (30.8)
A heavy drinker	85 (20.4)	70 (23.7)	15 (12.5)
A binge drinker	23 (5.5)	16 (5.4)	7 (5.8)
Dependency variables			
Not able to stop drinking once started			
No	147 (35.3)	82 (27.7)	65 (54.2)
Yes	269 (64.7)	214 (72.3)	55 (45.8)
Failed to function normally due to drinking			
No	197 (47.4)	125 (42.2)	72 (60.0)
Yes	219 (52.6)	171 (57.8)	48 (40.0)
Needed a drink first thing in the morning			
No	138 (33.2)	83 (28.0)	55 (45.8)
Yes	278 (66.8)	213 (72.0)	65 (54.2)
Social impact variable			
Problem with friends			
No	207 (49.8)	135 (45.6)	72 (60.0)
Yes	209 (50.2)	161 (54.4)	48 (40.0)
Health consequences variables			
Visited the hospital due to drinking			
No	337 (81.0)	232 (78.4)	105 (87.5)
Yes	79 (19.0)	64 (21.6)	15 (12.5)
Intent to quit			
No	125 (30.1)	87 (29.4)	38 (31.7)
Yes	291 (69.9)	209 (70.6)	82 (68.3)
Ever stopped drinking due to health reasons			
No	296 (71.2)	208 (70.3)	88 (73.3)
Yes	120 (28.9)	88 (29.7)	32 (26.7)

### Prevalence of illicit alcohol consumption

**Figure 2:**
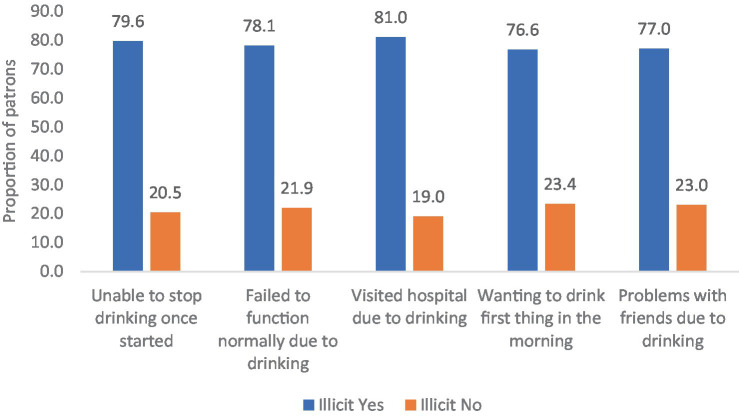
Patron reporting experiencing various socio-economic and health problems.

The prevalence of illicit alcohol consumption was 71.2%. This rate is higher for males (75.3%) compared to females (54.7%). The prevalence of illicit consumption was highest among employed individuals (88.5%) Among respondents with incomes less than K4,001, as well as those ranging between K4,001 and K10,000, the percentages of illicit alcohol consumption were 69.6 and 22.0%, respectively, (Table 1). More than 44.9% of the patrons who consumed alcohol daily reported consuming illicit alcohol. Among respondents who admitted to consuming illicit alcohol, a considerable number reported experiencing various socio-economic and health problems. These problems included visiting the hospital due to drinking incidents (81.0%), problems with friends (77.0%), a desire to drink first thing in the morning (76.6%), difficulty functioning normally (78.1%), and an inability to stop drinking once started (79.6%) (Figure 2).

### Factors associated with illicit alcohol consumption

Results from the univariate logistic regression analyses showed several factors as being significantly associated with consuming illicit alcohol among patrons in Zambia. These factors included gender, marital status, income levels, frequency of alcohol consumption, drinking pattern, inability to stop drinking once started, failure to function normally due to drinking and needing a drink first thing in the morning (Table 2). The multivariable logistic regression analysis conducted to explore factors associated with illicit alcohol consumption among youths in Zambia yielded several significant findings. After adjusting for various variables in the regression analysis, it was observed that females had an adjusted odds ratio (AOR: 0.40, 95% CI: 0.20–0.73, *p* = 0.003), indicating a decreased likelihood of engaging in illicit alcohol consumption. Additionally, residing in Ndola was associated with decreased odds of illicit alcohol consumption compared to Livingstone (AOR) 0.28 (95% CI: 0.12–0.62, *p* = 0.002) Individuals with incomes above K10,000 demonstrated lower odds of engaging in illicit alcohol consumption (AOR: 0.40, 95% CI: 0.19–0.85, *p* = 0.017). Moreover, individuals who consumed alcohol 1–2 days a month had an 62% lower likelihood of engaging in illicit alcohol consumption compared to daily drinkers (AOR: 0.38, 95% CI: 0.17–0.85, *p* = 0.018). Similarly, individuals who consumed alcohol 2–3 days a month and about 1 day a month or less often had reduced odds of engaging in illicit alcohol consumption by 81% (AOR: 0.19, 95% CI: 0.06–0.53, *p* = 0.002) and 99% (AOR: 0.01, 95% CI: 0.00–0.19, *p* = 0.001) respectively. Furthermore, those who reported dependency on alcohol, indicated by an inability to stop drinking, exhibited significantly increased odds of illicit alcohol consumption (AOR: 2.48, 95% CI: 1.35–4.57, *p* = 0.003).

**Table 2:** Factors associated with illicit alcohol consumption among patrons in Zambia.

Characteristics	*UOR (95% CI)	*p*-value	**AOR (95% CI)	*p*-value
Socio-demographic				
Age group				
18–29	Ref		Ref	
30–44	0.95 (0.59–1.54)	0.843	0.65 (0.34–1.24)	0.190
45–59	0.88 (0.46–1.67)	0.695	0.59 (0.25–1.40)	0.229
60–69	0.76 (0.13–4.34)	0.762	0.48 (0.05–5.00)	0.541
70 and above	0.38 (0.05–2.81)	0.345	0.78 (0.01–2.26)	0.183
Gender				
Male	Ref		Ref	
Female	0.40 (0.24–0.65)	**0.000**	0.38 (0.20–0.73)	**<0.003**
Marital status				
Married	Ref		Ref	
Not married	0.86 (0.55–1.33)	0.496	0.59 (0.32–1.06)	0.078
Separated	1.85 (0.67–5.12)	0.234	0.94 (0.29–3.04)	0.914
Employment status				
Employed	Ref		NA	
Unemployed	0.55 (0.29–1.03)	0.063		
Student	0.75 (0.18–3.05)	0.686		
Income				
Less than 4,001	Ref		Ref	
4,001 – 10,000	1.87 (1.00–3.52)	0.052	1.93 (0.90–4.17)	0.093
Above 10,000	0.44 (0.24–0.81)	**0.009**	0.40 (0.19–0.85)	**<0.017**
Residence				
Livingstone	Ref		Ref	
Lusaka	0.37 (0.20–0.68)	**0.002**	0.27 (0.12–0.56)	**<0.001**
Ndola	0.33 (0.18–0.62)	**0.001**	0.28 (0.12–0.62)	**<0.002**
Behavioral variables				
Age at first drink				
15<	Ref		Ref	
15–19	1.55 (0.89–2.72)	0.124	1.43 (0.72–2.83)	0.310
20–25	0.74 (0.40–1.36)	0.328	0.52 (0.24–1.17)	0.116
>25	0.70 (0.32–1.53)	0.372	0.71 (0.24–2.05)	0.519
Education				
Primary school (G1-G7)	Ref		Ref	
Secondary school (G8-G9)	0.99 (0.52–1.87)	0.972	0.59 (0.25–1.41)	0.239
High school (G10-12)	1.40 (0.77–2.57)	0.272	0.83 (0.35–1.95)	0.667
College/ University	0.79 (0.39–1.60)	0.511	0.51 (0.19–1.34)	0.171
First alcohol introducer				
Friend or acquaintance	Ref		NA	
Brother or sister	1.66 (0.46–5.93)	0.438		
Parent	0.88 (0.39–2.01)	0.768		
Spouse or Partner	0.27 (0.07–1.02)	0.054		
Other relative	0.61 (0.21–1.72)	0.347		
Stole it	0.51 (0.24–1.09)	0.082		
Purchased it myself	0.70 (0.38–1.28)	0.245		
Cannot recall	0.28 (0.13–1.09)	0.072		
Frequency of alcohol consumption				
Everyday	Ref		Ref	
5–6 days a week	1.00 (0.32–3.17)	1.000	0.97 (0.25–3.70)	0.959
3–4 days a week	0.63 (0.33–1.20)	0.160	1.01 (0.43–2.36)	0.984
1–2 days a week	0.32 (0.18–0.57)	**0.000**	0.38 (0.17–0.85)	**0.018**
2–3 days a month	0.18 (0.08–0.42)	**0.000**	0.19 (0.06–0.53)	**<0.002**
About 1 day a month	0.03 (0.00–0.25)	**0.001**	0.01 (0.00–0.19)	**<0.001**
Less often	0.26 (0.07–1.04)	0.057	0.14 (0.02–0.81)	**<0.028**
Alcohol pattern				
An occasional drinker	Ref		Ref	
A light drinker	1.70 (0.87–3.31)	0.119	1.69 (0.68–4.19)	0.259
A social drinker	1.24 (0.62–2.46)	0.543	0.99 (0.40–2.42)	0.976
A heavy drinker	3.01 (1.36–6.65)	**0.006**	1.38 (0.44–4.29)	0.577
A binge drinker	1.47 (0.52–4.22)	0.469	0.62 (0.16–2.45)	0.496
Dependency variables				
Cannot stop drinking				
No	Ref		Ref	
Yes	3.08 (1.99–4.79)	**0.000**	2.48 (1.35–4.57)	**<0.003**
Failed to function normally due to drinking				
No	Ref		Ref	
Yes	2.05 (1.33–3.16)	**0.001**	1.27 (0.71–2.25)	0.423
Needed a drink first thing in the morning				
No	Ref		Ref	
Yes	2.17 (1.40–3.37)	**0.001**	0.72 (0.38–1.37)	0.314
Social impact variable				
Had problems with friends due to drinking				
No	Ref		Ref	
Yes	1.79 (1.16–2.75)	**0.008**	0.92 (0.53–1.62)	0.784
Health consequence variables				
Visited hospital due to drinking				
No	Ref		Ref	
Yes	1.93 (1.05–3.55)	**0.034**	1.10 (0.51–2.39)	0.805
Intent to quit				
No	Ref		NA	
Yes	1.15 (0.73–1.83)	0.544		
Ever stopped drinking due to health reasons				
No	Ref		NA	
Yes	1.16 (0.72–1.87)	0.532		

Variables included in the multivariable analysis despite lack of statistical significance in univariate analysis: age group, marital status, education and age at first drink; UOR* Unadjusted odds ratio; AOR** Adjusted odds ratio; AOR. Bold values indicates statistically significant.

## Discussion

This study aimed to estimate the prevalence of illicit alcohol consumption and examine the socio-demographic and behavioral characteristics associated with illicit alcohol consumption among patrons in Zambia. Overall, almost 72% of patrons reported consuming illicit alcohol as well. Gender, marital status, cities/residences, income, frequency of alcohol consumption and not being able to stop drinking were significantly associated with illicit alcohol consumption.

The World Health Organization (WHO) estimated that 25% of all alcohol consumed is illicit, with the highest consumption occurring in LMICs (21, 22). In Zambia, 27% of participants interviewed consumed *tujilijili* or *junta*, while 18% consumed *kachasu*, together, these low-cost, high-ABV spirits account for 45% of illicit alcohol consumption (12). It is important to note that our results cannot be directly compared to those of the 2017 STEPS household survey due to the fact that the STEPS survey was a household survey that included participants who both consumed alcohol and those who did not (13), while our survey specifically interviewed people who already consume alcohol. However, our results clearly indicate a significantly high prevalence of illicit alcohol consumption in Zambia.

This is a public health concern considering that one-third of the Zambian population is relatively young and productive age group (23), who are supposed to be engaged in economic productivity. Illicit alcohol is more potent and addictive than regular alcohol (24), and people tend to be addicted and engage in vices that are detrimental to their health and society. Since these alcohols are unregulated, they contain toxic substances like methanol which causes organ damage, cardiovascular problems, neurological issues, and even death (25–27). Our study showed that more than 79% of the people visited the hospital due to a drinking incident and failed to function normally due to illicit alcohol. Additionally, people showed signs of alcohol dependency as they reported wanting a drink first thing in the morning wanting to drink. There is a need to address these factors holistically and communities can work toward safeguarding people’s health and well-being from the impact of illicit alcohol.

In the context of numerous studies that have demonstrated an association between male gender and alcohol consumption (28–30), it is not surprising that more than half of males are more likely to report illicit alcohol consumption than females. A potential reason is the gendered consequences of stigma, as it has been found that women who drink alcohol are perceived to lack morals and are considered sexually promiscuous, even going so far as being labeled sex workers regardless of their actual profession (29). Additionally, marriage was associated with illicit alcohol consumption despite not reaching statistical significance. It’s well known that marital status is a robust predictor of alcohol consumption but the direction of the causal relationship in some studies is not clear (31–33). In our study, those who were married were more likely to be involved in illicit alcohol consumption than single persons. This is contrary to what other studies have found where they have shown that the transition into marriage predicted a decrease in alcohol consumption across genders and age (33–35), It is not surprising however that our study found the contrary, a plausible explanation could be that people are coping with stress that comes with socio-economic life.

After adjusting for socio-demographic, behavioral psychological, and dependency variables, our analyses showed that those who consumed alcohol regularly every day were more likely to consume illicit alcohol. This pattern of drinking is associated with injuries, stroke, and cardiovascular deaths (36, 37). Studies have shown that there is an association between income and alcohol consumption in general. Those with disposable income tend to consume more alcohol than the less affluent (38) and are more likely to be frequent drinkers presumably because they can afford it and potentially have more social opportunities that include alcohol (39). However, in terms of illicit alcohol consumption there is an affordability and accessibility dynamics. Our study has shown that those with an income bracket less than k4,000 were more likely to consume illicit alcohol than those with income K4,000 – K10,000. The illicit alcohols especially home-distilled products like *kachasu, tujilijili, junta* are very cheap and readily accessible to a wider section of the people in the country. These unregulated production and sales of illicit alcohol such as kachasu, tujilijili and junta, poses serious health risks, undermines public health efforts, and increases alcohol-related diseases and deaths. Furthermore, illicit alcohol markets lead to loss of tax revenue for the government, harm legitimate businesses, and are mostly linked to criminality that destabilizes communities. Despite the Zambian government enacting laws to regulate alcohol through the liquor licensing bill of 2011 and the national alcohol policy of 2018, more needs to be done in terms of enforcement of these regulations.

To address these issues, stricter regulations and penalties for illicit alcohol production and sales can deter illegal activities by increasing fines and conducting regular inspections by the city council authorities and the Zambian Police. Additionally, public education campaigns about the dangers of illicit alcohol can reduce demand by informing people of the dangers of illicit consumption and legal consequences. Furthermore, business programs providing alternatives to illicit alcohol production can address root causes. The community programs that give alternative recreational activities, skills and job training would assist the young people and lastly, enhancing healthcare access for those affected by illicit alcohol can mitigate its impacts by offering treatment for addiction and related health issues.

### Strengths and limitations

Our study had several limitations. Firstly, due to the nature of our study design, we were unable to assess changes in the factors over time. Secondly, as an observational study, we could only identify factors associated illicit alcohol consumption, but could not establish causal relationships. Although we adjusted for known confounders in our multivariable model, there could still be residual confounders that are inherent in observational studies, which could potentially impact the interpretation of our study outcomes. Furthermore, the data in our study was based on self-reported responses, consuming illicit alcohol is often associated with poverty and economic hardship, as it is usually cheaper than regulated alcohol, usually this can lead to social exclusion and discrimination. This stigma can introduce the possibility of information bias and misclassification of illicit alcohol consumption leading to under reporting of the actual prevalence. Additionally, it is important to note that our study was conducted as a cross-sectional study in Zambia, and therefore its generalizability to other national or international settings may be limited hence further studies are needed from various factors, including social, cultural, and health outcomes related studies.

Despite these limitations, our study holds several strengths. Notably, it is the first-ever study to examine illicit alcohol consumption and its associated risk factors among the patrons in Zambia. By focusing specifically on this topic, we contribute valuable insights into an underexplored area of research. Additionally, our study utilized appropriate statistical methods, including adjustment for known confounders, to enhance the validity of our findings.

## Conclusion

This study identified sociodemographic, behavioral, and psychological factors associated with illicit alcohol consumption among patrons in Zambia. These findings underscore the need for implementing evidence-based, multi-level interventions. Such interventions could include educational programs, policy changes, community initiatives, and improved access to healthcare services. Effective and sustainable implementation in Zambia will require collaboration among healthcare professionals, policymakers, educators, and other community stakeholders, particularly parents and the young people at risk.

## Data Availability

The raw data supporting the conclusions of this article will be made available by the authors, without undue reservation.
